# Influence of PLDLA on the Rheological Properties of Regenerated Silk Fibroin-Based Gels

**DOI:** 10.1021/acs.biomac.5c01312

**Published:** 2026-01-23

**Authors:** Bianca Sabino Leocádio Antunes, Daniel Komatsu, Pâmela Soto Garcia, Cedric Dicko, Eliana Aparecida de Rezende Duek

**Affiliations:** † Post-graduation Program in Materials Sciences (PPGCM), Federal University of São Carlos (UFSCar), Sorocaba, São Paulo 18052-780, Brazil; ‡ Biomaterials Laboratory, Medical and Health Sciences Faculty, Pontifical Catholic University of São Paulo (PUC-SP), Sorocaba Campus, Sorocaba, São Paulo 18060-030, Brazil; § Pure and Applied Biochemistry Laboratory, Department of Chemistry, 5193 Lund University, Lund SE-221 00, Sweden

## Abstract

Regenerated silk fibroin (RSF), obtained from the silk fibers of the species *Bombyx mori*, is a promising biomaterial due to its excellent mechanical and biological properties, as well as presenting versatility for applications in tissue engineering. This is particularly evident through the development of scaffolds via the 3D printing process. The viscosity of RSF solutions can be controlled by adding other synthetic polymers, thereby ensuring improved mechanical and rheological properties and enhanced quality of printed structures. The objective of this study is to investigate the rheological properties of gels based on RSF and PLDLA (poly­(l-*co*-d,l-lactic acid)). The study involved the synthesis of PLDLA and the extraction of RSF from the silk threads of *B. mori*. Subsequently, RSF-PLDLA solutions in formic acid were prepared with varying concentrations, and subsequently subjected to rheological tests under steady-state and oscillatory regimes.

## Graphical abstract

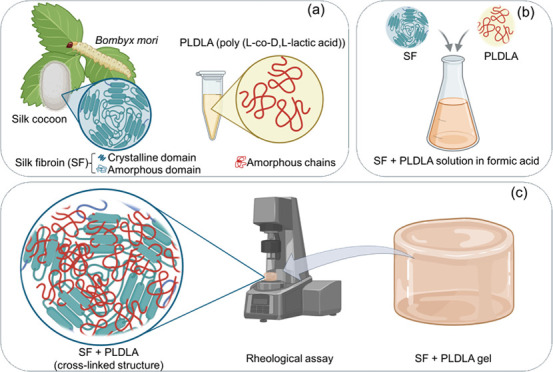

## 1 Introduction

Silkworms (*Bombyx mori*) produce cocoons from a fascinating protein-based material known as silk fibroin (SF). Silk fibroin is part of the soluble glycoprotein group and consists of light chain (L) and heavy chain (H) polypeptides.^1,2^ The H chain (390 kDa) has an amino acid sequence Gly–Ala–Gly–Ala–Gly–Ser, which leads to the formation of antiparallel β-sheet secondary structures through hydrogen bonds and hydrophobic interactions along the protein chain, which confer crystallinity and mechanical strength to the silk threads.^3,4^

Silk fibroin was highlighted as a biomaterial in 1993 by the Food and Drug Administration (FDA) and has since been widely used in clinical, biomedical, and tissue engineering applications.^5–7^ Regenerated silk fibroin (RSF) is the most suitable for applications as biomaterials and involves some steps such as degumming, dissolution, dialysis, and centrifugation.^8^ In addition, it can be obtained in the form of films, nanofibers, hydrogels, and sponges, from aqueous solution or organic solvent,^9,10^ through electrospinning^11,12^ and 3D printing.^13,14^

However, it is known that the entire process of obtaining RSF directly impacts its mechanical and rheological properties in comparison to the native SF, mainly because it involves many steps until reaching the final RSF.^15,16^ Holland et al. (2007)^17^ conducted a study of the rheological properties of native SF and RSF from the *B. mori* species, showing that RSF presented significantly reduced viscosity and storage modulus values (*G*′ ∼ 10^–1^ Pa) when compared to native SF (η ∼ 104) (*G* ∼ 102 Pa).

This decrease in mechanical properties can negatively affect when it is intended to subject RSF to subsequent processing techniques. Studies that develop RSF-based inks report difficulties in the 3D printing process, mainly when using low concentrations of RSF, due to the low viscosity of the system, resulting in difficulties in process reproducibility, as well as if used in high concentrations it can cause clogging in the printer nozzle. To overcome these problems, several studies have used RSF mixed with other polymers such as PVA,^18^ PEG,^19,20^ PLLA,^21^ PEO, and PVP^22^ aiming to induce the formation of a silk β-sheet structure, to improve mechanical and rheological properties.

Poly­(l-*co*-d,l-lactic acid), PLDLA, is a copolymer of the polyester family, with amorphous, biocompatible, biodegradable, and bioresorbable characteristics. This copolymer (PLA) originated from lactic acid and is a synthetic and aliphatic polyester, the target of many studies in biomedical applications.^23,24^ PLA exists in two stereochemical forms: poly­(l-lactic acid) (PLLA) and poly­(D,l-lactic acid) (PDLLA). PLLA is a polymer widely studied in biomedical applications.^21,25,26^ PLDLA is a copolymer that combines the mechanical characteristics of PLLA without the long degradation time required by this homopolymer, due to its high crystallinity.

This is possible because poly­(D,l-lactic acid), on the other hand, has a higher degradation rate, despite having mechanical properties lower than those of poly­(l-lactic acid).^27,28^ Due to these characteristics, PLDLA may be suitable for 3D printing of scaffolds, combining its characteristics with natural polymers such as silk fibroin to compose ink formulations.

## 2 Materials and Methods

### 2.1 Materials

PLDLA (*M*_w_ = 383,000 g/mol), in a 70/30 ratio of l-lactic acid to d,l-lactic acid, was synthesized using the methodology described in the literature by Motta and Duek (MOTTA; DUEK, 2007). Silk fibroin (SF) was used as received. Sodium carbonate (Sigma-Aldrich), lithium bromide (Sigma-Aldrich), formic acid (>88%, Synth, Brazil), and Dialysis tubing cellulose membrane (Sigma-Aldrich - Typical molecular weight cutoff: 12.000 Da) were purchased. All reagents were used as received.

### 2.2 Methods

#### 2.2.1 Preparation of RSF

RSF was extracted from silk threads of the species *B. mori* following an extraction protocol.^29,30^ The silk fibers (5 g) were cut into small pieces and added to 1 L of aqueous Na_2_CO_3_ solution (0.5 wt %) at 60 °C, stirred for 1 h and then the fibers were washed abundantly with distilled water. The degumming process was repeated once more to effectively remove the sericin. After this process, the fibers were dried in an oven at 50 °C for 24 h and then 2.5 g of dry silk fibers were dissolved in 25 mL of lithium bromide solution at 70 °C, under magnetic stirring for up to 40 min until a slightly viscous solution with a transparent and yellowish appearance was obtained. The solution was cooled to room temperature and transferred to cellulose membranes and dialyzed against distilled water for 72 h, with 2 water changes per day. After this time, the solution was centrifuged at 4500 rpm for 30 min at 4 °C. Finally, the fibroin solution was frozen and lyophilized for 48 h to obtain the RSF.

#### 2.2.2 Preparation of RSF, PLDLA and RSF-PLDLA Solutions

RSF and PLDLA solutions were prepared in different concentrations varying the RSF (wt %); PLDLA (wt %), and tested in the rheological assays to select the most suitable one to compose the RSF/PLDLA solution. In the next step, the RSF (and PLDLA) was solubilized in formic acid, under constant stirring, for 72 h at 25 °C. Table 1 illustrates the RSF concentrations used in the preparation of the solutions.

**Table 1:** Solutions with Different Concentrations of RSF and PLDLA

	concentrations (wt %)							
RSF	0.75	1.00	1.50	3.00	5.00	7.00	10.00	15.00
PLDLA	-	1.00	-	3.00	5.00	7.00	10.00	15.00

After performing the rheological analyses of the RSF solutions, the solution selected to continue the tests, with the most suitable properties, was 15.00 wt % (RSF15). Thus, this was chosen to be used in the formulation of the RSF/PLDLA solutions, which was obtained by incorporating 1.00, 5.00, 10.00, and 15.00 wt % of PLDLA when compared to the mass of RSF. PLDLA was added to the RSF solution with constant stirring for 24 h at 25 °C. The solutions were refrigerated at 4 °C until their use. Table 2 illustrates the concentrations of PLDLA added to the RSF15 to prepare the RSF/PLDLA solutions.

**Table 2:** Concentrations of PLDLA Added to the RSF15 to Prepare the RSF/PLDLA Solutions

RSF (wt %)	15.00				
PLDLA (wt %)	0.00	1.00	5.00	10.00	15.00

### 2.3 Rheological Parameters

The rheological characterization of all solutions was performed using a DHR-2 Rheometer (TA Instruments). The tests were conducted at 25 °C using a cone-plate geometry with a cone angle of 2°0′32″, a diameter of 40 mm, and a truncation gap of 55 μm. Initially, steady-state measurements were carried out to obtain viscosity curves by varying the shear rate between 0.001 and 1000 s^–1^. Subsequently, oscillatory tests were performed, including amplitude and frequency sweep tests. For the amplitude test, the strain rate was varied between 0.01% and 100% at a constant angular frequency of 10 rad/s to determine the linear viscoelastic region (LVE) of the gel and the yield point (transition from solid-like behavior at rest to liquid-like behavior at the onset of flow). The strain value within the linear viscoelastic region (LVE) was then used for the frequency sweep test, which ranged from 0.1 to 240 rad/s, to measure the loss modulus (*G*″) and storage modulus (*G*′).

## 3 Results and Discussion

### 3.1 Specific Viscosity (η_sp_) as a Function of RSF Concentrations

Initially, a preliminary study of pure RSF and PLDLA solutions (Figure S2 Supporting Information) was conducted. Figure 1 shows the double logarithmic plot of specific viscosity, as shown in eq 1

1 $$\eta_{Sp}=\frac{\eta -\eta_{s}}{\eta_{s}}$$

where η_Sp_ is the specific viscosity, η is the viscosity of the referred solution and η_s_ is the solvent viscosity. Concentration of the RFS solutions (wt %) were: 0.50; 0.75; 1.00; 1.50; 3.00; 5.00; 7.00; 10.00; and 15.00 wt %. A dependence of the zero-shear viscosity in function the increase in RSF concentration was observed, where a transition from the dilute to the semidilute regime could be identified.^31^ The eq 2, as referred by Verbeeten (2010),^32^ derived from the models proposed by Tuinier et al. (1999)^33^ and Ren et al. (2003)^34^ were applied. It suggests that zero-shear viscosity as a function of concentration can be described by a combination of two concentration-dependent terms with different slopes, corresponding to the dilute and semidilute regime.

2 $${\eta_{0}\left(C\right)=\eta }_{S}+\left(\eta_{r,2}-\eta_{S}\right)\left\{{k_{1}\left(\frac{C}{C_{r,1}}\right)}^{n1}+{\left(\frac{C}{C_{r,2}}\right)}^{n2}\right\},k_{1}\frac{\eta_{r,1}-\eta_{S}}{\eta_{r,2}-\eta_{S}}$$

where η_0_ is the zero-shear viscosity, *C* is the concentration, η_S_ is the solvent viscosity. The parameters related to 1 and 2 correspond to the dilute and semidilute regimes, respectively, η_*r*,1_ and η_*r*,2_ represent the zero-shear viscosities at the reference concentrations *C*_*r*,1_ and *C*_*r*,2_, while the exponents *n*1 and *n*2 are the slopes on a double logarithmic scale.

**Figure 1:**
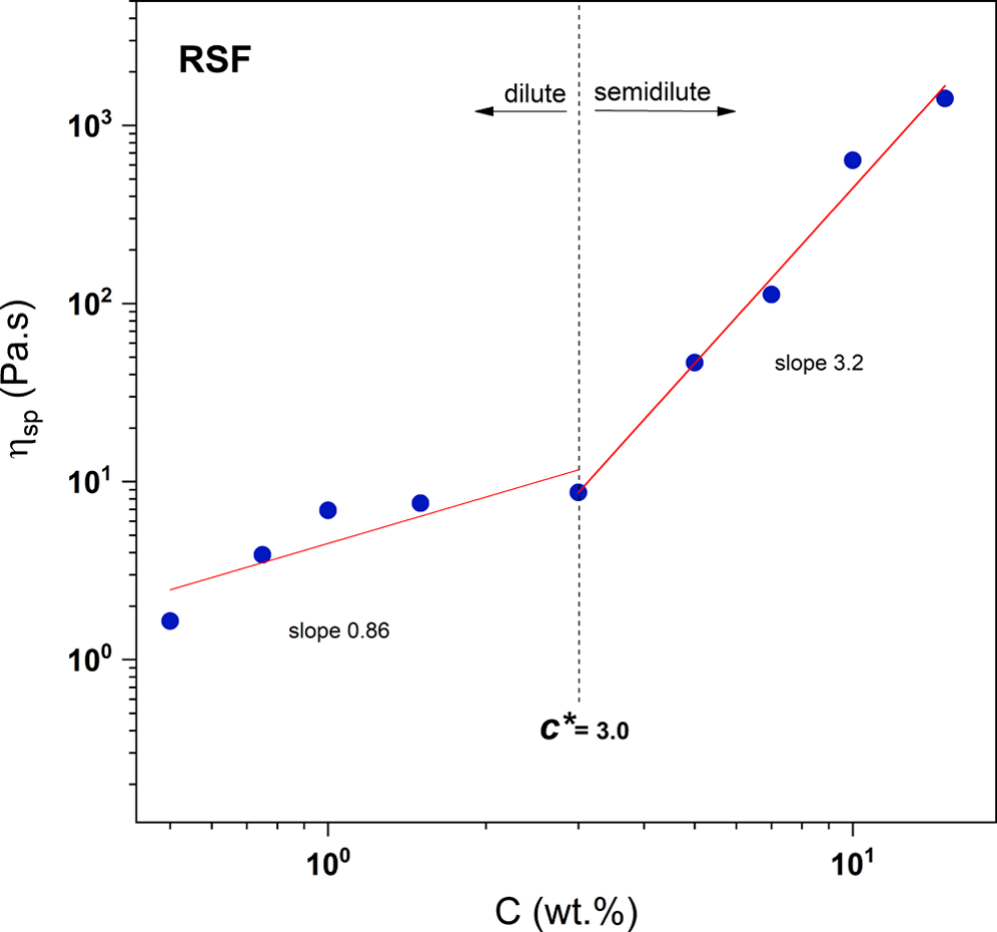
Dependence of specific viscosity as a function of RSF concentrations (wt %): 0.50; 0.75; 1.00; 1.50; 3.00; 5.00; 7.00; 10.00; and 15.00 (wt %).

According to the physical scaling law^35,36^ at the concentration *C*_*r*,1_ in the dilute regime, the polymer chains are individual and exhibit little to no interaction among themselves, with the relaxation time being independent of concentration, leading to an exponent *n*1 = 1. In contrast, in the semidilute regime (*C*_*r*,2_) the interaction and entanglement of the chains increase, such that the zero-shear viscosity η_*r*,2_ becomes time-dependent, represented by the linear relaxation spectrum $\eta_{r,2}=\sum_{i=1}^{M}=Gi\lambda i$ . Following the method described in the literature by Verbeeten (2010)^32^ and Yu (2013)^37^ a double logarithmic plot of specific viscosity as a function of concentration was generated and compared with simulation results based on eq 2.

In this case, two distinct regions were observed, with slopes of η_sp_ ∼ *C* ^0.86^ and η_sp_ ∼ *C* ^3.2^, corresponding to the diluted and semidiluted regimes, respectively.^37,38^ It is known that the RSF contains a minor fraction of charged amino acids, which can impart weak polyelectrolyte character to the chains. Thus, although the presence of charged amino acids in RSF may slightly reduce the observed scaling exponents, the values obtained in this study are broadly consistent with the physical scaling law for neutral polymers,^35,39,40^ where the thresholds are η_sp_ ∼ *C* ^1.0–1.5^ and η_sp_ ∼ *C* ^3.5–4.5^. The transition between these regimes was characterized by a critical concentration (C*), equivalent to 3.00 wt % RSF. When values are lower than 3.00 wt %, the system remains in the diluted regime; when values are above, the system transitions to the semidiluted regime. In the diluted regime, the polymer chains are individual and exhibit greater freedom of movement relative to the solvent.

Consequently, viscosity increases linearly with increasing concentration until the critical concentration C*, also known as the overlap concentration, is reached. At this point, the number of individual protein chains increases, leading to interactions between them. As concentration increases, entering the semidiluted regime, the first signs of chain entanglement appear. To better understand the entanglement effects, the plateau modulus (*G*_e_) parameter was employed, as presented on eq 3 ^41^

3 $$G_{e}=\frac{\eta_{0}}{\lambda_{sr}}$$

where λ_sr_ is the relaxation time (reciprocal of the shear rate at which shear thinning begins), and η_0_ is the zero-shear viscosity.

Figure 2 shows the double logarithmic plot of the plateau modulus as a function of RSF concentration. For RSF concentrations up to 5.00 wt %, consistent plateau modulus values describing entanglement effects were not observed. According to Zhang et al. (2015)^42^ and Colby (2010),^39^ the scaling law exponent describing the effect of entanglement for polymer solutions (neutral polymers in a good solvent) is 2.31 (*G*_e_ ∼ *C* ^2.31^). The red dashed line includes only RSF concentrations approaching the values within the entanglement region.

**Figure 2:**
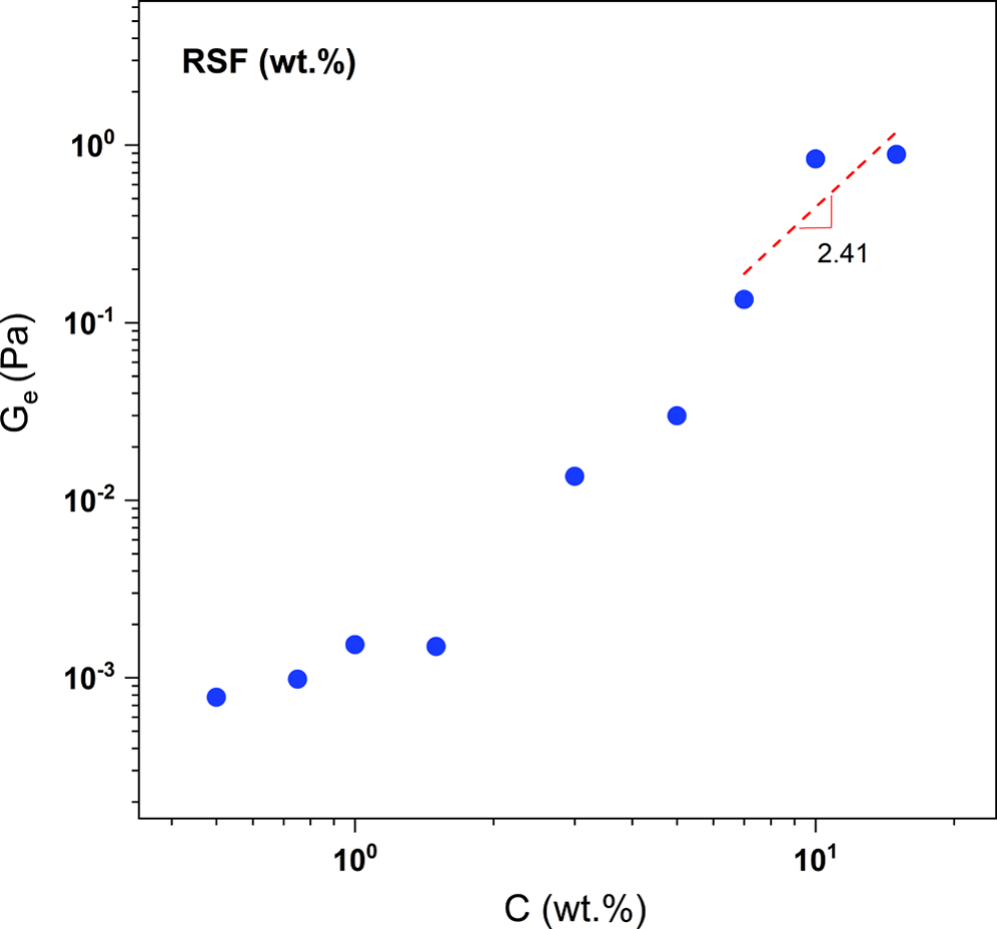
Dependence of plateau modulus as a function of RSF concentrations (wt %): 0.50; 0.75; 1.00; 3.00; 5.00; 7.00; 10.00; and 15.00.

As observed in Figure 2 and based on the calculated *G*_e_ values, RSF chain entanglements began at a concentration of 7.00 wt %, as the slope of the line for the last three points (7.00, 10.00, and 15.00 wt % RSF) was found to be 2.41. In this case, chain entanglements in RSF solutions at concentrations higher than 7.00 wt % occur due to the increased presence of antiparallel beta (β) sheets, which are associated with higher RSF concentrations, the secondary protein conformation of RSF, and the entanglement of other primary structures such as alpha helices and/or random coils. Consequently, this results in a gradual and continuous increase in the viscosity of the gels for samples with 7.00, 10.00, and 15.00 wt % RSF.

### 3.2 Steady-State Viscosity of RSF Solutions

Figure 3 illustrates the viscosity curves as a function of the shear rate for RSF solutions (Figure S1 Supporting Information). According to the curves, RSF concentrations up to 3.00 wt % exhibited a shear-thinning behavior at low shear rates, followed by Newtonian plateaus extending to infinite shear rates. As the RSF concentration increased, this behavior shifted to a slight decrease in viscosity with increased shear rate. The pseudoplastic behavior became more pronounced starting at a concentration of 5.00 wt %, except for the 10.00 wt % RSF sample. For this sample, a change in curve behavior was observed, displaying Newtonian plateaus at low shear rates, followed by viscosity decreases at higher shear rates. The well-defined pseudoplastic regions suggest that the increased RSF concentration resulted in a higher prevalence of antiparallel beta sheets, which are associated with crystalline regions.^38,43,44^ The formation of these structured networks requires the application of high shear rates to achieve chain disentanglement and alignment in the flow direction.

**Figure 3:**
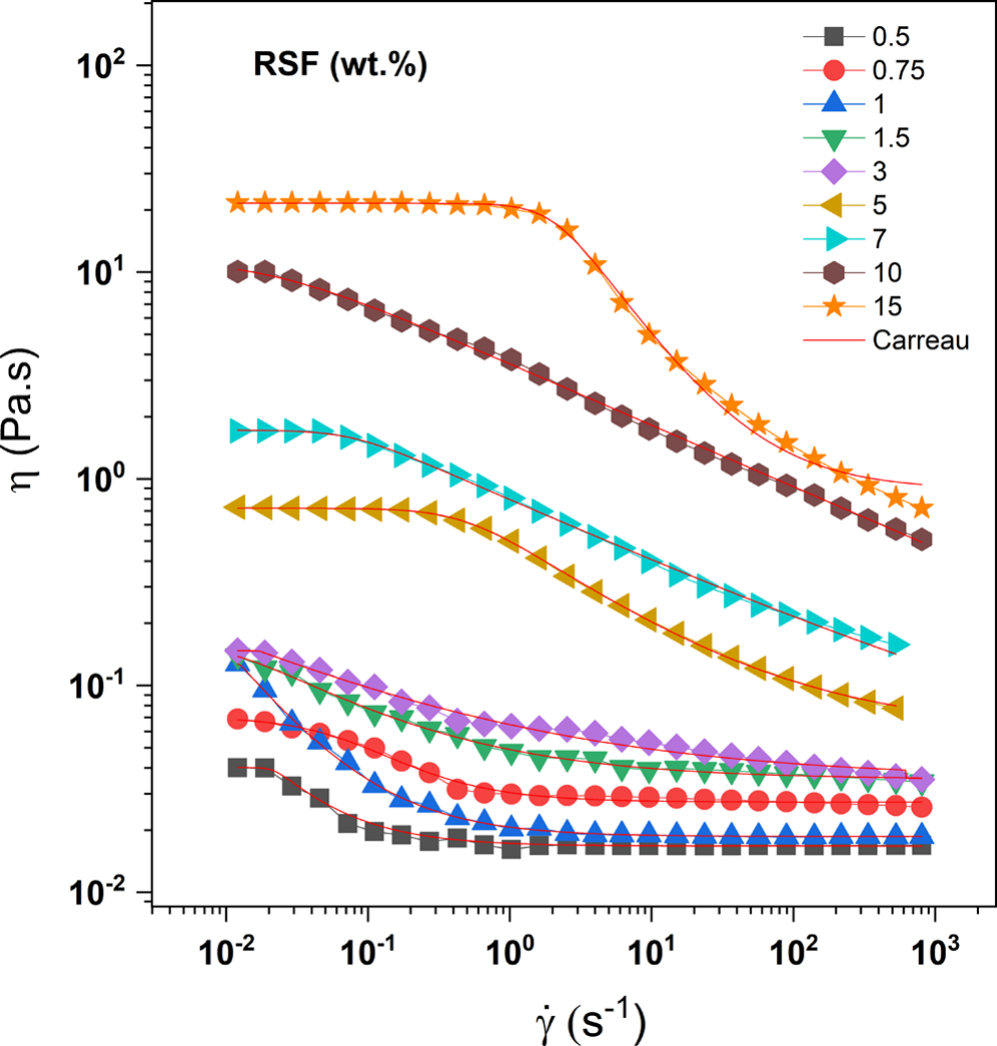
Viscosity curves for RSF solutions at different concentrations (wt %): 0.50, 0.75, 1.00, 1.50, 3.00, 5.00, 7.00, 10.00, and 15.00. The red line corresponds to the fit using the Carreau model (1972).

In addition to generating the viscosity curves, the Carreau model (1972)^45^ was applied as a fitting approach. This model describes shear-thinning behavior and viscosity plateaus at infinite shear rates, specifically for low-concentration solutions (eq 4)­

4 $$\frac{\eta \left(\mathrm{\gamma ̇}\right)-\eta_{\infty }}{\eta_{0-}\eta_{\infty }}={\left[{1+\left(\lambda \gamma \right)}^{2}\right]}^{n-1/2}$$

where η_0_ is the zero-shear viscosity, η_∞_ is the infinite-shear viscosity, γ̇ is the critical shear rate associated with the relaxation time λ, and *n* represents the power-law index. The model provided a good fit for the distinct curve behaviors as the RSF concentration increased. The fitting parameters for each concentration are presented in Table 3.

**Table 3:** Fitting Parameters to the Carreau Model for the Viscosity Curves with Different RSF Concentrations

concentration (wt %)	η_0_ (Pa s)	η_∞_ (Pa s)	γ̇ _c_ (s^–1^)	*n*	*R* ^2^
0.50	0.04018	0.01675	0.02907	0.95441	0.98571
0.75	0.07041	0.02731	0,01771	0.94539	0.99406
1.00	0.12802	0.01861	0.01801	0.86190	0.99031
1.50	0.16321	0.04193	0.01871	0.68279	0.99165
3.00	0.14727	0.35139	0.04571	0.67145	0.99887
5.00	0.72197	0.06086	0.27198	0.62851	0.99981
7.00	1.72110	0.03129	0.11143	0.67272	0.99912
10.00	9.69699	0.11735	0.02097	0.66188	0.99965
15.00	11.5339	0.61484	0.98717	0.62177	0.99989

### 3.3 Viscoelastic Properties of RSF Solutions

To investigate the viscoelastic properties of RSF solutions, frequency sweep tests of the *G*′ and *G*″ moduli were performed as a function of angular frequency. Figure 4 shows the oscillatory regime curves for solutions with 1.0, 3.0, 5.0, 7.0, 10.0, and 15.0 wt % RSF (Figure S3 Supporting Information). It was observed that the 1.0, 3.0, and 5.0 wt % concentrations exhibited a characteristic viscous behavior, showing a liquid-like response (*G*″ > *G*′) at low frequencies, transitioning to a predominantly elastic behavior (*G*′ > *G*″) at high frequencies due to protein chain entanglement. The frequency values at the crossover point (*G*′ = *G*″) increased with increasing concentration.^38,46^

**Figure 4:**
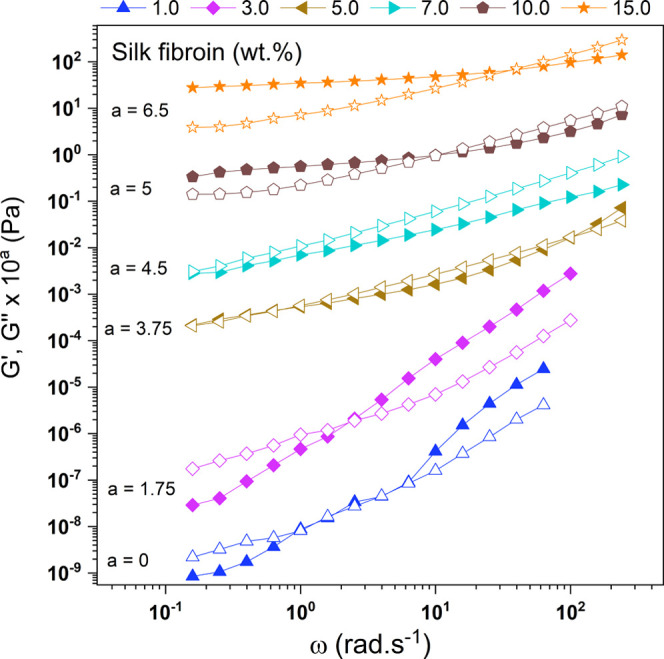
Storage modulus (*G*′, filled symbols) and loss modulus (*G*″, open symbols) curves as a function of angular frequency for RSF solutions: 1.00, 3.00, 5.00, 7.00, 10.00, and 15.00 wt %. The data were vertically shifted to avoid overlapping.

A behavior change was observed for the 7% RSF concentration, with *G*″ > *G*′ as the angular frequency increased. This behavior suggests the formation of physical interactions, resulting in a weak gel. This may occur because higher concentrations cause the protein chains to resist increasing oscillatory shear at lower frequencies, transitioning from predominantly solid behavior at rest (*G*′ > *G*′) to a predominantly viscous behavior (*G*′ > *G*′). For the 7% concentration, the transition from *G*′ > *G*′ to *G*′ > *G*′ occurs early in the frequency range (10–1), while for the 10.0% and 15.0% concentrations, it occurs at higher frequency values. This fact occurs because in the most concentrated ones, the physical network that forms the gel is more cohesive.^10^ Thus, for continuing the rheological analyses with the addition of PLDLA, the 15% RSF concentration was chosen. This concentration exhibits more suitable rheological characteristics (viscosity and viscoelasticity), enabling a better interaction response when PLDLA is added due to its more structured organization.

### 3.4 Steady-State Viscosity of RSF15_PLDLA Solutions

Figure 5 shows the viscosity curves of the RSF 15.00% (RSF15) solution with different concentrations of PLDLA. For all concentrations of PLDLA added to the RSF15 solution, the formation of a Newtonian plateau and shear thinning behavior was observed. It was also observed that the increase in PLDLA concentration in the RSF15 gel was proportional to the increase in viscosity.

**Figure 5:**
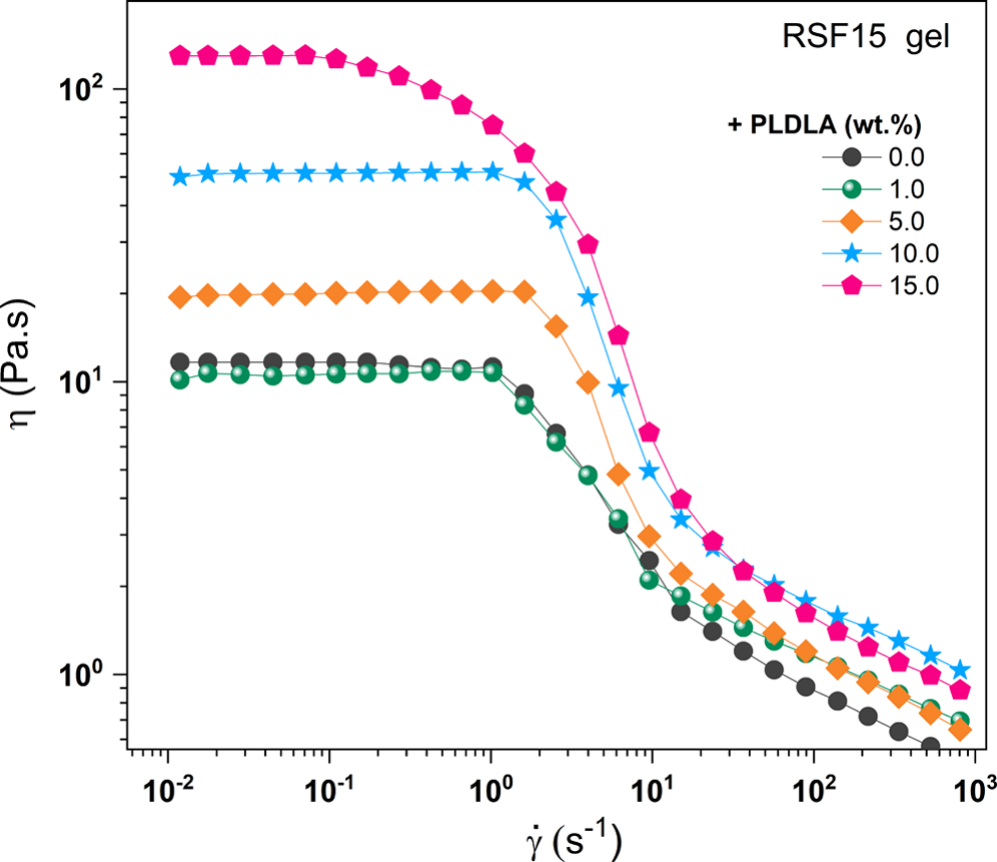
Viscosity vs shear rate for RSF15 samples with different PLDLA concentrations (0.0, 1.0, 5.0, 10.0, and 15.0 wt %).

The addition of 1.0% PLDLA (RSF15_PLDLA10) to the RSF15 solution caused minimal changes in viscosity and viscosity curve profile compared to the RSF15 sample. However, a significant increase in viscosity was observed for the RSF15_PLDLA15 sample compared to RSF15, with a well-defined pseudoplastic region between 10^–1^ and 10^3^ s^–1^. This behavior suggests stronger interactions between the PLDLA chains and the β-sheet crystals of the RSF15 sample. These interactions likely resulted in the formation of a cross-linked network through hydrogen bonding, restricting the movement of the RSF and PLDLA structures. Consequently, a substantial increase in viscosity was observed compared to the RSF15 sample.^39,47,48^

With the addition of PLDLA 15 to the RSF15 solution, a reduction in the Newtonian plateau was observed compared to the RSF15 solution without PLDLA, allowing the gel to flow at lower shear rates. This behavior can be advantageous for processes such as injection molding, as it requires lower injection force and facilitates flow. This decrease may have occurred due to rearrangements of the PLDLA polymer chains with the beta (β) sheet structures of RSF, resulting in the disorganization of the chains. The presence of PLDLA promotes the formation of free chains that move between the antiparallel beta (β) sheets of RSF, resulting in a decrease in viscosity values.^19^

### 3.5 Viscoelastic Properties of RSF15_PLDLA Solutions

Figure 6 illustrates the storage and loss moduli of RSF15 samples with the addition of different concentrations of PLDLA. In this case, the RSF15_PLDLA1 sample exhibited parallel storage (*G*′) and loss (*G*″) moduli with minimal dependence on frequency increases.

**Figure 6:**
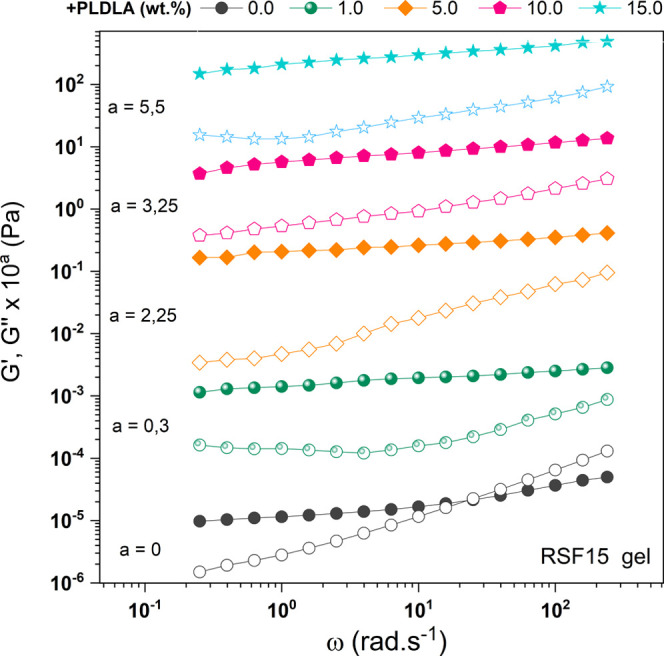
Storage modulus (*G*′, solid symbols) and loss modulus (*G*″, open symbols) curves as a function of angular frequency for RSF solutions with PLDLA concentrations of 0.0, 1.0, 5.0, 10.0, and 15.0 wt %. Data were vertically shifted to avoid overlapping.

Conversely, all other samples (RSF15_PLDLA5, RSF15_PLDLA10, and RSF15_PLDLA15) showed a higher storage modulus compared to the loss modulus (*G*′ > *G*″), indicating gel formation, with *G*′ values increasing as PLDLA concentration increased. This suggests that the addition of PLDLA to the RSF15 solution induced the formation of antiparallel beta-sheets, resulting in a gel with predominantly elastic characteristics. This behavior is attributed to the increased interaction between PLDLA chains and the beta-sheet (β) crystals of RSF, restricting the mobility of these structures due to the presence of cross-linked networks.^49,50^ To complement those findings, the flow types and the corresponding chain structures of the samples were analyzed using LAOS, as presented in the Supporting Information.

Figure 7 shows the damping factor curves (tan δ = *G*″/*G*′) as a function of the angular frequency for RSF15 samples with different PLDLA concentrations (0.0, 1.0, 5.0, 10.0, and 15.0 wt %). This test evaluates the behavior of a sample, which can either exhibit a liquid-like behavior, indicating a viscous character (tan δ > 1), or a solid-like behavior, with a predominantly elastic character (tan δ < 1).^51,52^

**Figure 7:**
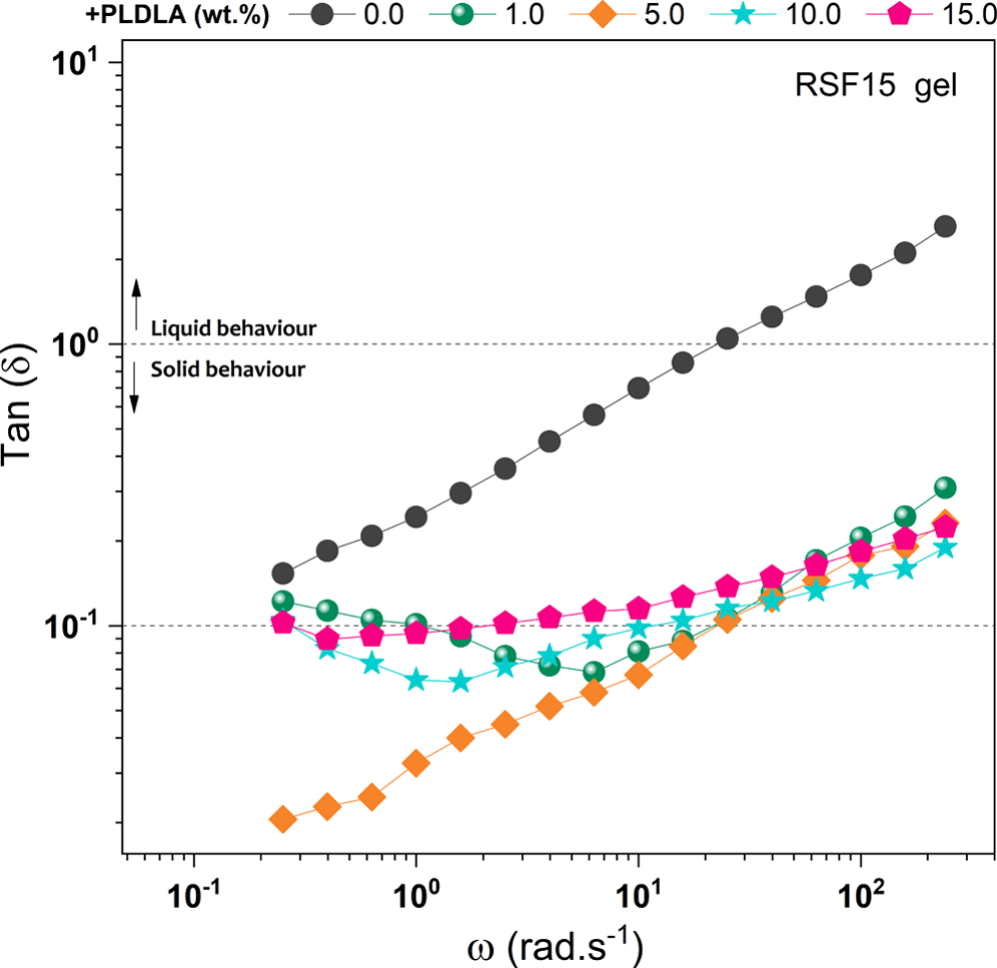
Damping factor (tan δ) as a function of angular frequency for RSF solutions with PLDLA additions: 0.0, 1.0, 5.0, 10.0, and 15.0 wt %.

It was observed that RSF15 without PLDLA addition (RSF15_PLDLAϕ) showed greater variation in tan δ values with increasing frequency, starting with tan δ < 1 at lower frequencies and gradually increasing until reaching tan δ > 1 at frequencies higher than 10^1^ rad s^–1^. Upon adding PLDLA to the RSF15 sample, a behavior change was observed compared to RSF15_PLDLAϕ, with little variation in tan δ values as frequency increased. This indicates that the addition of PLDLA promoted gel formation. All PLDLA concentrations exhibited tan δ values close to the threshold for forming more resistant gels (tan δ < 10^–1^). Therefore, this suggests that the addition of PLDLA to the RSF15 sample may have induced the formation of antiparallel beta (β) sheets, resulting in more structured gels.^22,53^

The gel point for the RSF samples (with or without PLDLA) can be observed through the damping factor. The Kramers–Krönig relationship states that the tangent of the loss angle δ is independent of frequency at the gel point^51,54^ (eq 5)­

5 $$\tan\delta =\frac{G^{\prime \prime }}{G^{\prime }}=\tan\left(\frac{n\pi }{2}\right)$$

The gel point is defined by the relaxation modulus *G*(*t*) and was proposed by Winter and Chambon (1986)^55^ as eq 6

6 $$G\left(t\right)=St^{-1}$$

where *S* is the gel strength and *n* is the relaxation exponent (0 < *n* < 1). At the gel point, an equilibrium of the dynamic mechanical properties occurs, described by a power law relationship between the moduli and frequency (eq 7)­

7 $$G^{\prime }\left(\omega \right)\alpha G^{\prime \prime }\left(\omega \right)\alpha \omega^{n}$$

where the storage (log *G*′) and loss (log *G*″) moduli are parallel as a function of angular frequency (log ω), with *n* representing the slope of the curves. Thus, the tangent of the loss angle (tan δ) is independent of angular frequency at the gel point.

Figure 8a (ω = 0.25 to 6.3 rad s^–1^) and Figure 8b (ω = 10 to 240 rad s^–1^) show the damping factor tan δ as a function of PLDLA concentration. In Figure 8a, a transition from liquid-like to solid-like behavior was observed upon the addition of 1.00 wt % PLDLA to the RSF15 sample (RSF15_PLDLA1) compared to the RSF15 sample (RSF15_PLDLAϕ). The crossover point, where frequency dependence was no longer observed, occurred at a concentration of 0.70 wt %, in the solid domain (tan δ < 1), near the line of formation of more resistant gels (tan δ < 10^–1^). For the RSF15_PLDLA5 sample, the tan δ values decreased significantly, with a large variation in tan δ values as the angular frequency increased. This behavior is consistent with what is shown in Figure 6, where the *G*′ and *G*″ moduli vary with increasing frequency. At the concentrations of 10.00 and 15.00 wt % PLDLA, the tan δ values were close to 10^–1^, indicating gel formation. Additionally, the tan δ values did not vary significantly with increasing frequency, which may indicate a trend toward becoming more frequency-independent.

**Figure 8:**
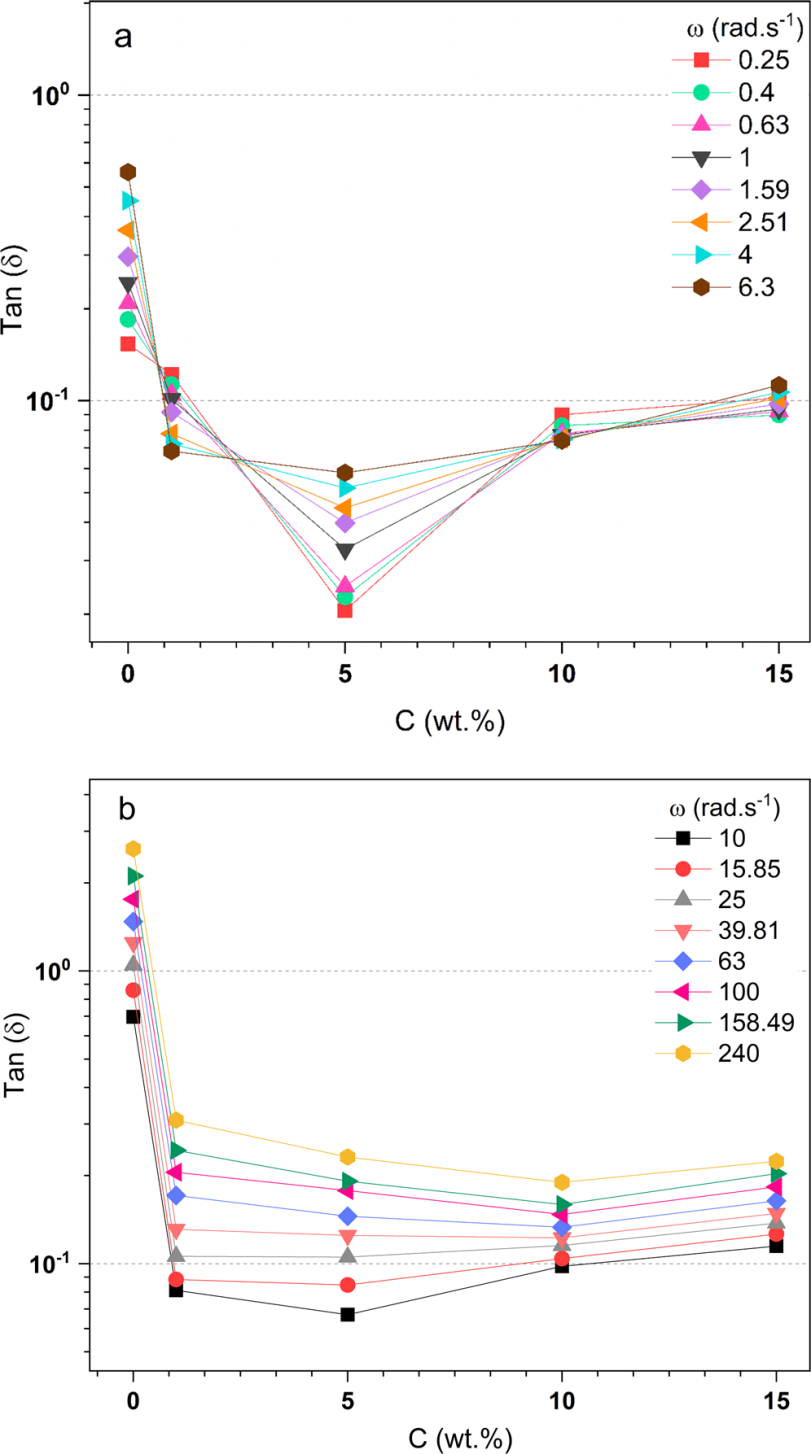
Damping factor as a function of concentration (wt %) of the RSF15 solution with PLDLA additions. The 0.0 wt % concentration represents the RSF solution without PLDLA addition (PLDLAϕ). The angular frequency increases from (a,b).

From the angular frequency value of 10.0 rad s^–1^ (Figure 8b), the values of tan δ increased with the angular frequency for all the solutions studied. This shows that the solutions respond to the increased oscillation, leading to the disentanglement of the polymeric structures. It was also observed that the values of tan delta shifted, approaching the liquid domain (RSF15_PLDLAϕ) and moving toward the solid domain with the increase in PLDLA concentration. Additionally, they exhibited reduced dependence on the angular frequency with increasing PLDLA concentration, indicating that the gels tend to resist oscillatory stress more, showing greater mechanical strength, even with frequency variations.

## 4 Conclusion

This study covered the investigation of RSF solutions at various concentrations, where the transition from the dilute to the semidilute regime was detected, along with a critical concentration C* (7.0 wt %) at which these regime changes occurred. The concentration of 15.0 wt % RSF (RSF15) was then selected for further experiments, varying the PLDLA concentrations. The viscosity curves as a function of shear rate showed that the addition of 15.0 wt % PLDLA significantly increased the viscosity value and exhibited a well-defined pseudoplastic region, suggesting ease in injection processes. In oscillatory tests, it was shown that the addition of PLDLA to the RSF15 solution altered its behavior by presenting parallel *G*′ and *G*″ moduli, indicating the formation of gels. These data were corroborated by the tan delta vs frequency graph, where RSF15_PLDLA solutions showed significantly reduced tan delta values, close to 10^–1^, with predominantly elastic characteristics. Thus, this study demonstrated that the addition of a synthetic polymer (PLDLA) improved the rheological properties of the RSF15 gel, indicating potential for 3D scaffold printing.

## Supplementary Material



## Notes

The PLDLA plots: viscosity curves, viscosity vs concentration, frequency sweep; and amplitude assay curves of RSF/PLDLA formulations (PDF)
