# Supplementary material for: Influence of PLDLA on the Rheological Properties of Regenerated Silk Fibroin-Based Gels
Source: Biomacromolecules. 2026 Jan 23;27(2):1187–95. doi: 10.1021/acs.biomac.5c01312 (PMC12892308; doi:10.1021/acs.biomac.5c01312)
Supplement: Supplementary file 1 [file bm5c01312_si_001.pdf]

Influence of PLDLA on the Rheological Properties of Regenerated Silk Fibroin-Based Gels

Bianca Sabino Leocádio Antunes (0000-0001-5273-5138)[1*], Daniel Komatsu (0000-0002-2441-8650)[2], Pâmela Soto Garcia 0000-0001-6131-0369[2,3], Cedric Dicko 0000-0001-6377-3500[3], Eliana Aparecida de Rezende Duek (0000-0001-6327-7363)[1,2*]

[1] Post-graduation Program in Materials Sciences (PPGCM), Federal University of São Carlos (UFSCar), Sorocaba, SP, 18052-780, Brazil

[2] Biomaterials Laboratory, Medical and Health Sciences Faculty, Pontifical Catholic University of São Paulo (PUC-Sorocaba), SP, 18060-030, Brazil

[3] Pure and Applied Biochemistry Laboratory, Department of Chemistry, Lund University (Lund SE-221 00, Sweden)

* Corresponding authors: **biancaleocadio@estudante.ufscar.br** and **eliduek@fem.unicamp.br**

Supporting Information

Below are graphs of tests performed with PLDLA as viscosity curves as a function of shear rate; Specific viscosity as a function of concentration; Frequency sweep tests

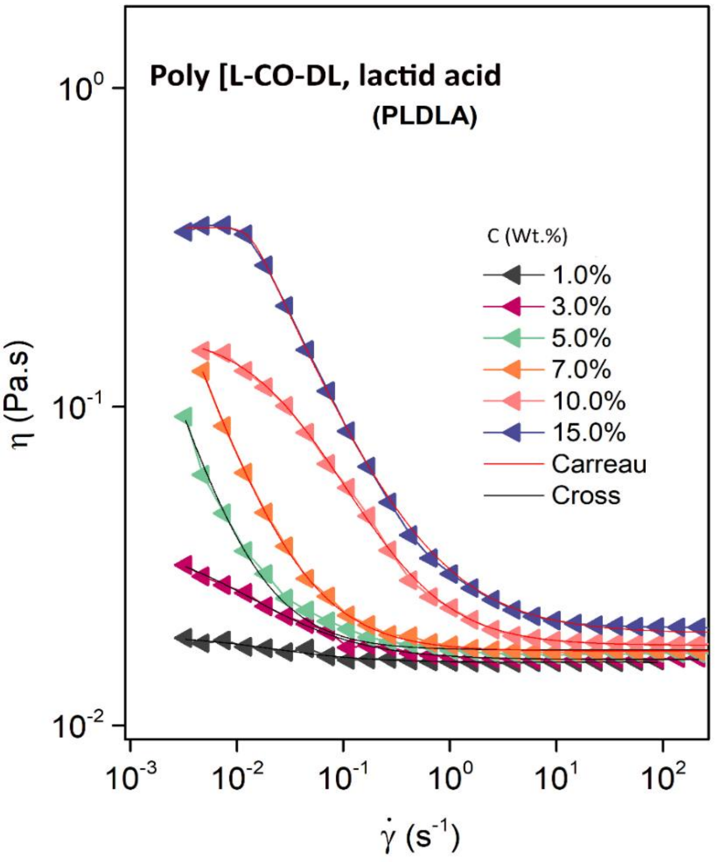

Figure S1. Viscosity curves for PLDLA solutions at different concentrations (wt.%): 1.0, 3.0, 5.0, 7.0, 10.0, and 15.0.

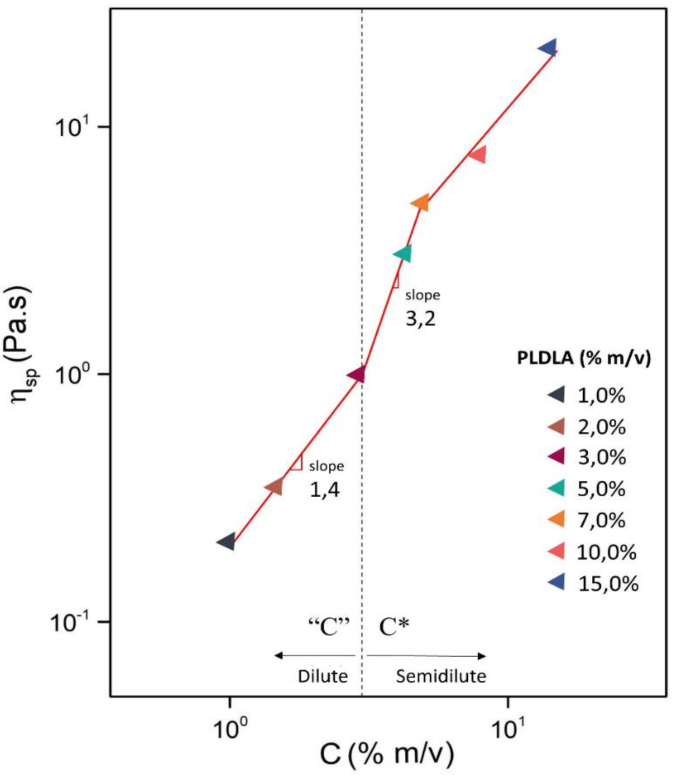

Figure S2. Dependence of Specific Viscosity as a function of PLDLA concentrations (wt.%): 1.0; 2.0; 3.0; 5.00; 7.00; 10.0; and 15.0 (wt.%).

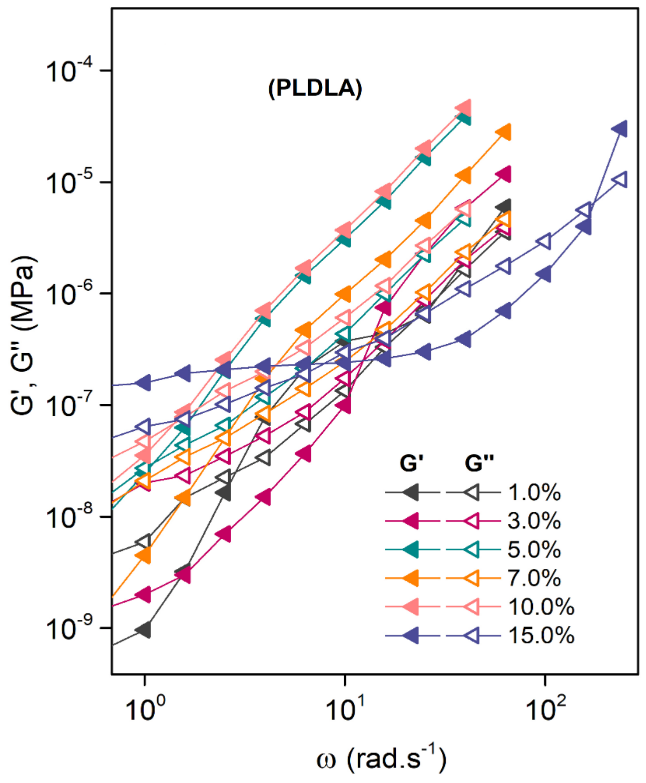

Figure S3. Storage modulus (G′, filled symbols) and loss modulus (G″, open symbols) curves as a function of angular frequency for PLDLA solutions: 1.0, 3.0, 5.0, 7.0, 10.0, and 15.0 wt.%.

Large Amplitude Oscillatory Shear

Flow types and the corresponding chain structures can be discussed in the following Figures. They are based on the patterns of large amplitude oscillatory shear (LAOS) behavior observed in the experiments. We have attempted to explain each pattern in terms of the interaction within the fluid system or shear-induced formation of the microstructure, if it exists. Therefore, the types of LAOS behavior may be classified into at least four types in non-linear region: type I, strain thinning (G', G" decreasing); type II, strain hardening (G' and G" increasing); type III, weak strain overshoot (G' decreasing, G" increasing followed by decreasing); type IV, strong strain overshoot (G', G" increasing followed by decreasing).

At low strains (up to 10%), the RSF solution exhibits a linear viscoelastic region (LVR), where fibroin chains interact through hydrogen bonding, forming a stable network. In this regime, the system responds linearly, meaning that the chains deform elastically without disrupting the network structure. In the intermediate strain range (10–100%), where G″ increases and reaches a maximum while G′ begins to decrease, chain misalignment occurs along with the partial rupture of hydrogen bonds between fibroin chains. Finally, at higher strains (above 100%), the physical fibroin network collapses as interchain associations break, leading to the loss of elastic connectivity. Therefore, the RSF solution exhibits type IV behavior - weak strain overshoot behavior.

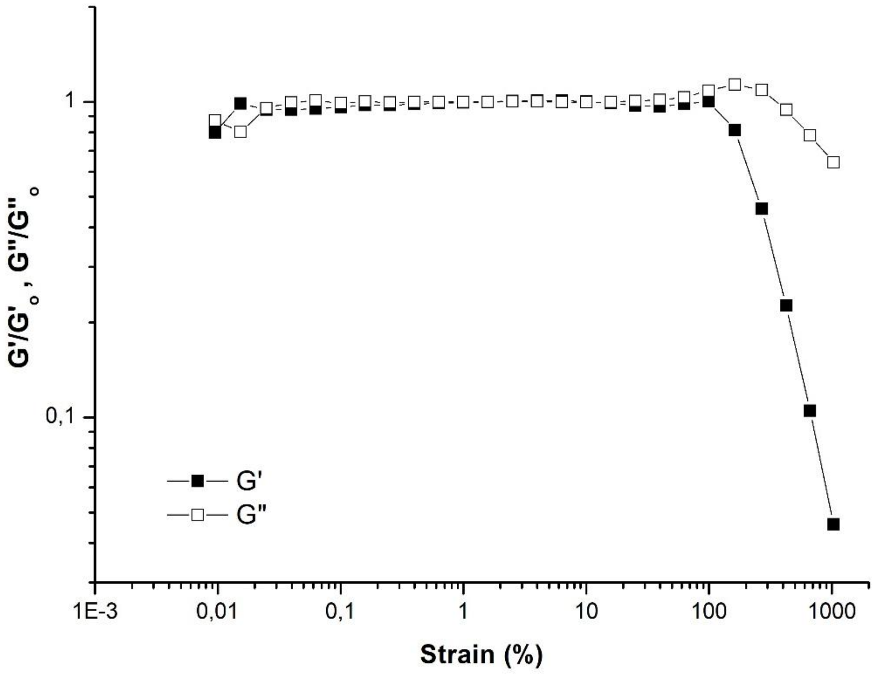

Figure S4. SRF solution - Reduced moduli G'/G'_0 and G''/G''_0 versus strain, where G_0 and G_0 are the moduli in the linear viscoelastic region.

RSF+PLDLA1% initially shows an increase in G` and G”. This shows that the fibroin and PLDLA chains are interconnected, resulting in efficient stress transfer that occurs through secondary interactions between PLDLA and RSF chains (hydrogen bonds). In this case, the chains begin to orient and align in the direction of deformation. Therefore, the RSF+PLDLA1% sample exhibits type IV behavior - strong strain overshoot, indicating a cohesive and partially compatible network, with good interfacial interaction and good resistance to initial deformation.

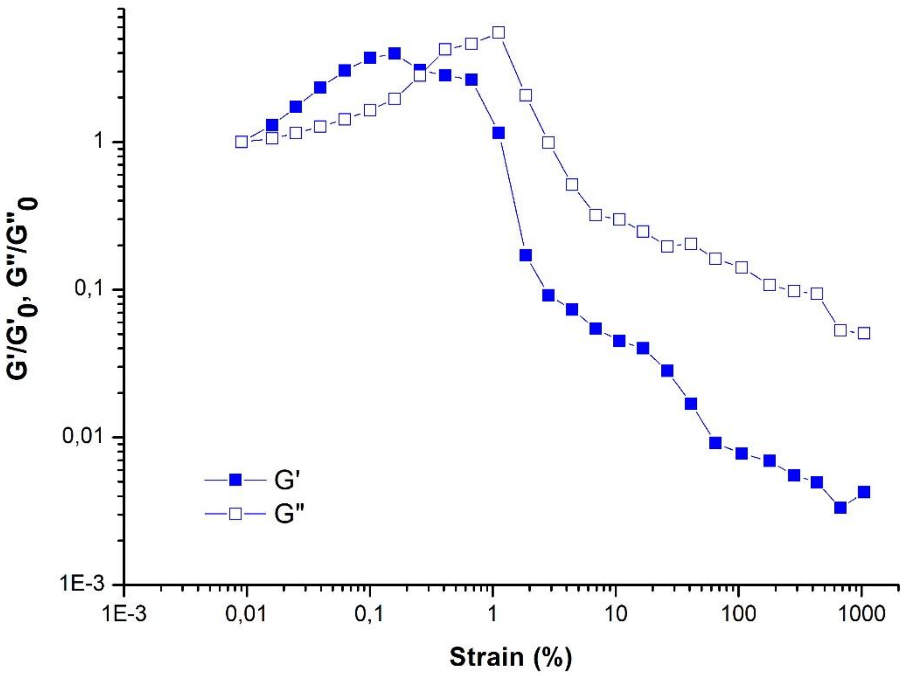

Figure S5. SRF+PLDLA1% - Reduced moduli G’/G’$_0$ and G”/G”$_0$ versus strain, where G$_0$ and G$_0$ are the moduli in the linear viscoelastic region.

For the RSF+PLDLA5% and RSF+PLDLA10% samples, the behavior is similar to that observed for the pure RSF solution; however, two distinct drops are observed in the G′ curve. This feature usually indicates multiple structural relaxation or network rupture processes occurring over different strain ranges. At low strains (<1%), both G′ and G″ remain nearly constant, meaning that the network formed between RSF and PLDLA chains remains intact, and the system behaves as a partially structured physical gel. In the strain range between 1% and 10%, the first drop in G′

appears, which is associated with the rupture of hydrogen bonds between PLDLA and amorphous fibroin chains. Additionally, amorphous PLDLA exhibits chain mobility that relaxes under lower stresses, contributing to this first decrease. Finally, in the strain range between 10% and 100%, a second drop in G′ is observed, occurring in the same region as the collapse of the pure RSF solution. This second decay corresponds to the breakdown of fibroin associations (β-sheets/aggregates) that persist up to higher deformations. For these samples (RSF+PLDLA5%, RSF+PLDLA7%, and RSF+PLDLA10%), the observed response is neither purely strain thinning nor purely overshoot, but rather a hybrid combination. Therefore, this behavior suggests an extended type IV behavior - weak strain overshoot behavior - in which the system stores elastic energy at two distinct structural levels and dissipates it in two successive stages.

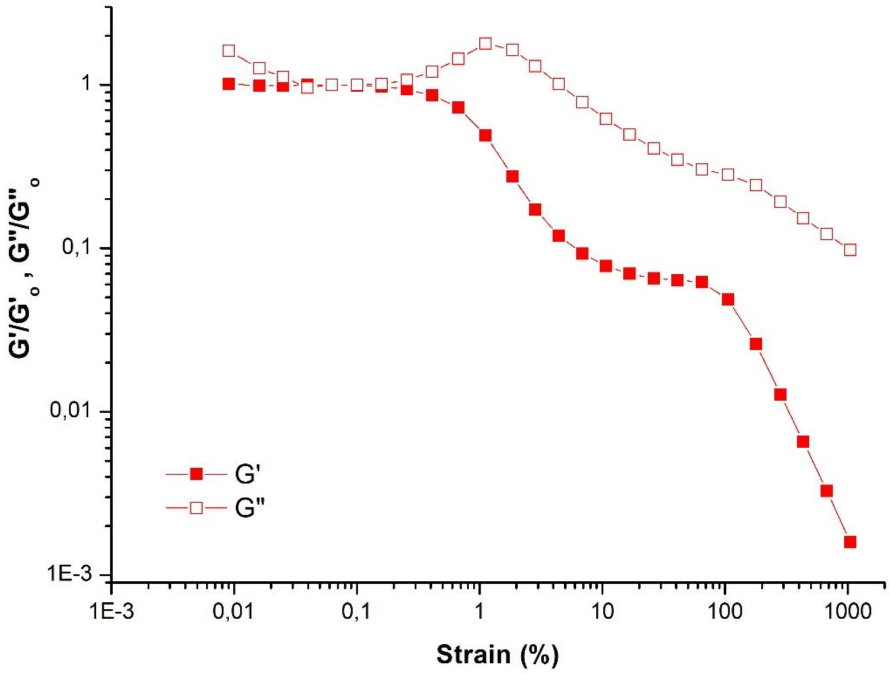

Figure S6. SRF+PLDLA5% - Reduced moduli G’/G’$_0$ and G”/G”$_0$ versus strain, where G_0 and G_0 are the moduli in the linear viscoelastic region.

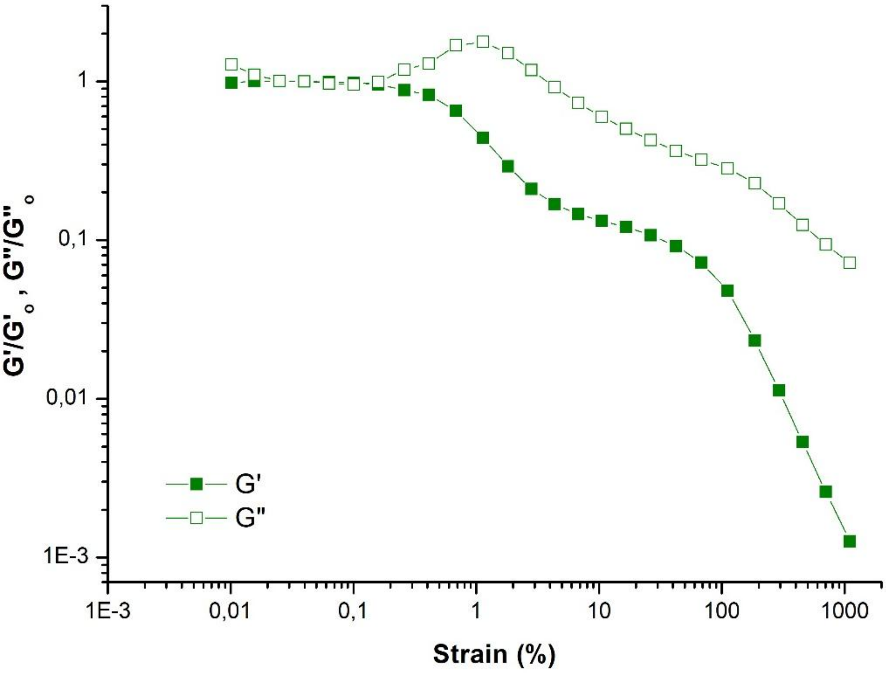

Figure S7. SRF+PLDLA10% - Reduced moduli G'/G'$_0$ and G"/G"$_0$ versus strain, where G_0 and G_0 are the moduli in the linear viscoelastic region.

The RSF+PLDLA15% sample, which contains the highest PLDLA concentration, exhibits only one drop in G′, similar to the pure RSF solution. At higher PLDLA contents, this phenomenon may result from the strong interactions between PLDLA and fibroin chains, leading to the formation of a single, more cohesive network with greater rigidity than that of samples with lower PLDLA concentrations (RSF+PLDLA5%, RSF+PLDLA7%, and RSF+PLDLA10%). Consequently, the structural collapse occurs within a strain range similar to that of the RSF solution. Therefore, the RSF+PLDLA15% sample exhibits type IV behavior - weak strain overshoot behavior - analogous to that of RSF solution.

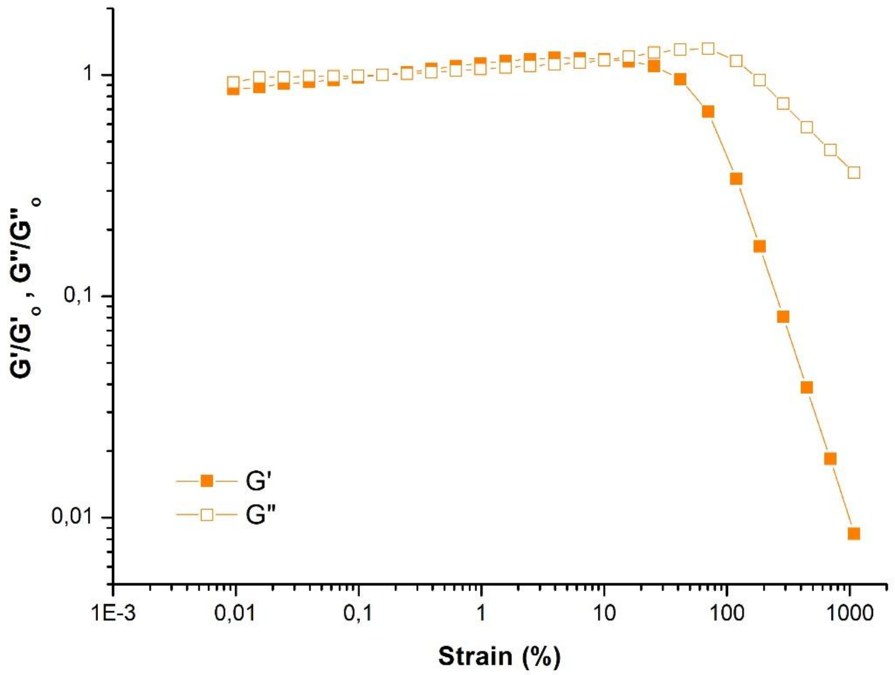

Figure S8. SRF+PLDLA15% - Reduced moduli G'/G'$_0$ and G"/G"$_0$ versus strain, where G$_0$ and G$_0$ are the moduli in the linear viscoelastic region.
